# Emerging Applications of Porphyrins and Metalloporphyrins in Biomedicine and Diagnostic Magnetic Resonance Imaging

**DOI:** 10.3390/bios8040095

**Published:** 2018-10-19

**Authors:** Muhammad Imran, Muhammad Ramzan, Ahmad Kaleem Qureshi, Muhammad Azhar Khan, Muhammad Tariq

**Affiliations:** 1 Department of Chemistry, Baghdad-Ul-Jadeed Campus, The Islamia University of Bahawalpur, Bahawalpur 63100, Pakistan; ak.qureshi@iub.edu.pk; 2 Department of Physics, Baghdad-Ul-Jadeed Campus, The Islamia University of Bahawalpur, Bahawalpur 63100, Pakistan; azhar.khan@iub.edu.pk; 3 Institute of Chemical Sciences, Bahauddin Zakariya University, Multan 60800, Pakistan; mtnazir@yahoo.com

**Keywords:** porphyrins, metalloporphyrins, photophysics, photochemistry, photodynamic therapy, photosensitizer, magnetic resonance imaging, contrast agents, bio-imaging, drug delivery

## Abstract

In recent years, scientific advancements have constantly increased at a significant rate in the field of biomedical science. Keeping this in view, the application of porphyrins and metalloporphyrins in the field of biomedical science is gaining substantial importance. Porphyrins are the most widely studied tetrapyrrole-based compounds because of their important roles in vital biological processes. The cavity of porphyrins containing four pyrrolic nitrogens is well suited for the binding majority of metal ions to form metalloporphyrins. Porphyrins and metalloporphyrins possess peculiar photochemical, photophysical, and photoredox properties which are tunable through structural modifications. Their beneficial photophysical properties, such as the long wavelength of emission and absorption, high singlet oxygen quantum yield, and low in vivo toxicity, have drawn scientists’ interest to discover new dimensions in the biomedical field. Applications of porphyrins and metalloporphyrins have been pursued in the perspective of contrast agents for magnetic resonance imaging (MRI), photodynamic therapy (PDT) of cancer, bio-imaging, and other biomedical applications. This review discusses photophysics and the photochemistry of porphyrins and their metal complexes. Secondly, it explains the current developments and mode of action for contrast agents for MRI. Moreover, the application of porphyrin and metalloporphyrin-based molecules as a photosensitizer in PDT of cancer, the mechanism of the generation of reactive oxygen species (ROS), factors that determine the efficiency of PDT, and the developments to improve this technology are delineated. The last part explores the most recent research and developments on metalloporphyrin-based materials in bio-imaging, drug delivery, and the determination of ferrochelatase in bone marrow indicating their prospective clinical applications.

## 1. Introduction

**Figure 1:**
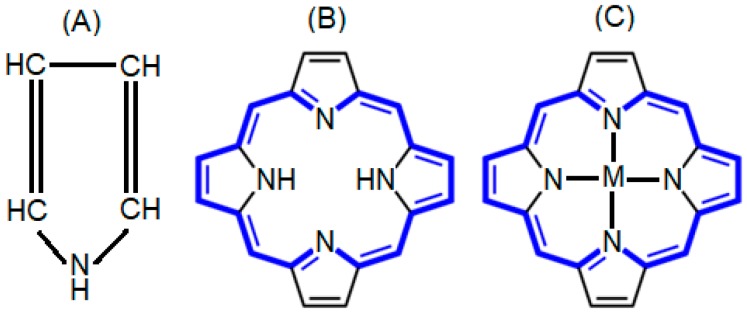
Typical structures, i.e., (**A**) pyrrole, and (**B**) porphyrin consist of four pyrrole rings joined by methene bridges, and (**C**) metalloporphyrin (M = Fe, Mn, Cr).

Porphyrins represent a unique class of heterocyclic tetrapyrrolic organic molecules which are the most ubiquitous compounds found in nature. These macrocycles derive their name from “porphura” which was used for the first time by ancient Greeks for the intense purple color [1]. From the structural viewpoint, porphyrin is composed of four pyrrolic units that are linked in a coplanar fashion by four methene bridges that give a planar macrocyclic structure to the porphyrin molecule (Figure 1). It has an extended conjugated 18 π-electron system which is responsible for its aromatic behavior, and its limited size cavity enables the accommodation of large metal cations [2,3]. Porphyrins are of fundamental significance on this planet to sustain life in various ways like storage and transport of oxygen, chlorophyll for photosynthesis, different types of enzymes and vitamins [4,5]. Synthetic porphyrins and metalloporphyrins have inspiring biological, photophysical, and photochemical properties and are promising candidates for diseases treatment [6], biological imaging [7], industrial [8]^,^ analytical [9], photocatalytic [10], nonlinear optics (NLO) [11], and molecular photovoltaics [12,13].

In the last few decades, researchers have expanded the use of porphyrin-based compounds for medical, drug delivery, bio-sensing, and bio-imaging purposes [14,15]. Porphyrin-based molecules have been pioneering theranostic agents not only for MRI, and photodynamic cancer therapy but also for drug delivery and single cell imaging [16,17,18,19]. Moreover, these compounds have potential applications as chemosensors [20]. Dong et al. [18] explored the application of the porphyrin-incorporated hydrogel with four arm-copolymer as promising drug carrier system. The clinical applications based on photophysical properties are embedded in the aromatic macrocyclic structure of porphyrins and metalloporphyrins [1]. In the free base porphyrins out of 22 π-electrons 18 are supposed to be conjugated, which are responsible for the characteristic redox and electronic properties. Some other compounds such as chlorin and bacteriochlorin show resemblances in structure with porphyrins [21]. Due to the conjugation of π-electrons on the frontier orbitals, porphyrins possess distinctive UV–VIS spectra because metalloporphyrins have four-fold symmetry and four nitrogen atoms directed towards the center of the porphyrin core [22]. Extensive conjugation of 18 electrons in porphyrins gives rise to facile π→π* transitions that give rise to two distinct bands within the visible region of electromagnetic radiations. An intense absorption band called B band or Soret band between 350 and 500 nm resulting from a ground state to second excited singlet state (S_0_→S_2_) with molar absorption coefficient 10^5^ M^−1^ cm^−1^ and a less intense band known as Q-band observed from 500 to 750 nm resulting from ground state to first excited state (S_0_→S_1_) with molar absorption coefficient 10^4^ M^−1^ cm^−1^ [23]. Insertion of a metal ion into the porphyrin cavity or the protonation of the nitrogen atoms or variation of the peripheral substituents may result in a change in the wavelength and intensity of the absorption spectrum [24].

Freebase porphyrins can form a complex with various metal cations and can adopt a wide variety of conformation: planar, domed, saddled, ruffled, etc. Depending on the size of coordinating metal cations, metalloporphyrins are of two types. If the cationic metal size of coordinating metal is 55–80 pm, in-plane metalloporphyrins are formed. In this type of metalloporphyrins, the metal centers are situated in the plane of porphyrin rings. In addition to the size of metal cations, the conformation of porphyrins also depends on additional factors, including the presence of axial ligands, the presence, and size of peripheral substituents, etc. If the cationic radius is greater than 80–90 pm out-of-plane or sitting-atop (SAT) metalloporphyrins are formed in which the metal atoms are located out of the porphyrin plane [3,4]. The photophysical, photochemical, and redox properties of the metalloporphyrins can be controlled by the fine-tuning of out-of-plane distance in the porphyrin cavity. By changing peripheral substituents and central metal can provide a broad diversity of biochemical functions. Different types of porphyrins and metalloporphyrins have been extensively examined and realized for biomedical and imaging applications [15,25,26,27].

Porphyrins and metalloporphyrins have a system of highly conjugated π bonds which helps in effectively absorbing visible light and, therefore, are promising photosensitizers. Upon light absorption, electrons of photosensitizer are transformed from ground to excited electronic state. After internal conversion, the excited electrons return to the ground state via fluorescence emission, or it may also non-radiatively decays back to the ground state without emitting a photon. It may also transform to a triplet state of a longer life after undergoing a non-radiative spin forbidden electronic transition. According to the literature, the longer lifetime of triplet state of the porphyrin allows to interact with the environment and produce ROS through different possible routes. In this review, the potential use of porphyrin and metalloporphyrins for biomedicine and diagnostic purposes has been discussed.

## 2. Photophysics and Photochemistry of Porphyrins and Metalloporphyrins

Photochemistry is related to the chemistry of excited electronic states. The absorption of a photon by a molecular species results in a change in electronic distribution and can induce a considerable alteration in physical and chemical properties of that molecule [28]. The photophysics, photochemistry and spectroscopic properties of porphyrins and metalloporphyrins, as well as their electronically excited states, remain a dynamic area of scientific research. Owing to their strong ability to absorb light, they have been extensively considered as diagnostic, therapeutic, and theranostic agents [29,30,31]. 

**Figure 2:**
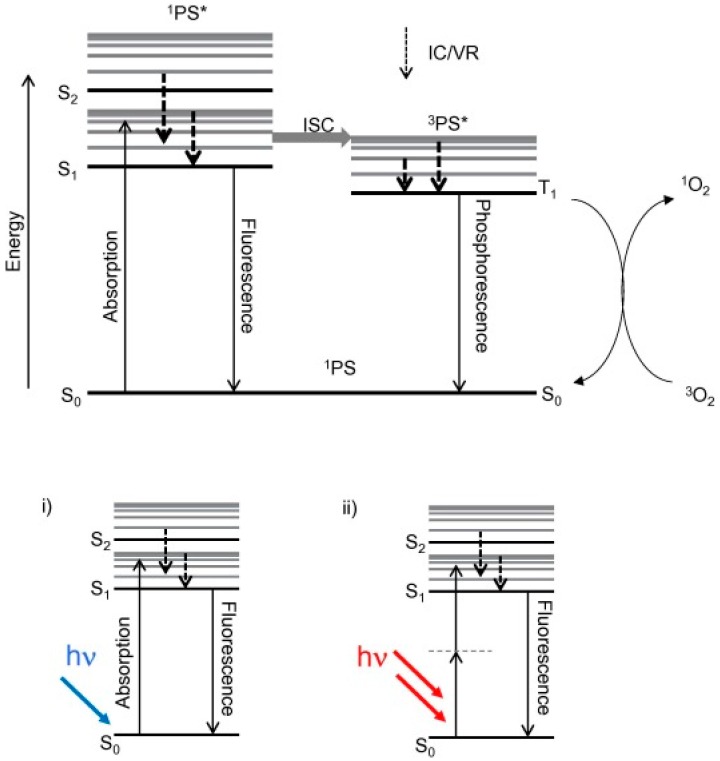
A simplified Jablonski diagram showing typical energy levels and transitions relevant to the formation of the triplet state of the photosensitizer and photosensitization of molecular oxygen. IC = internal conversion, VR = vibrational relaxation, ISC = intersystem crossing. Reproduced from McKenzie et al. [32], with permission from Elsevier.

The emission properties of porphyrins and their derivatives involve the relaxation of excited states to ground states takes place by photon emission. The Jablonski diagram has explained the mechanism of the excitation and relaxation processes in the molecules. Figure 2 illustrates a simplified Jablonski diagram [32]. The emission process starts from S_1_ this is known as Kasha’s rule, which is a lowest excited state, in case of a higher electronic excited state like S_2_, S_3_ and S_n_ a process of internal conversion take place before the emission [33]. Porphyrins in their singlet excited state may undergo relaxation from highest to lowest vibrational level. The relaxation between different vibrational levels can be slower than intersystem crossing and radiative routes. In addition to the biological importance of porphyrins and their derivatives, they are also exciting compounds from the non-radiative decay, fluorescence and phosphorescence perspective [33]. On excitation of porphyrins very minor part of the irradiation energy is wasted via heat dissipation, which is evident from the fact that the overall quantum yield of intersystem crossing and fluorescence in the realization of a triplet state is about 95%. Owing to this characteristic, porphyrins and their metal complexes are useful in photosensitization and various biomedical applications. Porphyrins exhibit two types of fluorescence, i.e., (1) S_1_-fluorescence and (2) S_2_-fluorescence. The first type S_1_-fluorescence is relatively strong and more widely studied in the range of 550–800 nm. A weak luminescence has also been observed at 400–550 nm upon excitation at the Soret or B band. The main reasons for this deviation from the Kasha’s rule are structural rigidity and the relatively large energy gap between the singlet-2 and singlet-1 excited states. Moreover, Raman and Rayleigh scattering of the solvents may disturb the specific detection of S2-fluorescence intensity. The energy of the emission band of metalloporphyrins is controlled by the size and electronic structure of the coordinated metal ion. A decrease in quantum yield of fluorescence and an increase in the rate of intersystem crossing has been observed when the metal ion gets coordinated to a porphyrin ring [4,5,34]. The unique properties of porphyrins and metalloporphyrins, especially very superior photochemistry and photophysics, make them promising candidates for biomedical applications [35].

## 3. Porphyrins and Metalloporphyrins as Contrasting Agents for Magnetic Resonance Imaging

**Figure 3:**
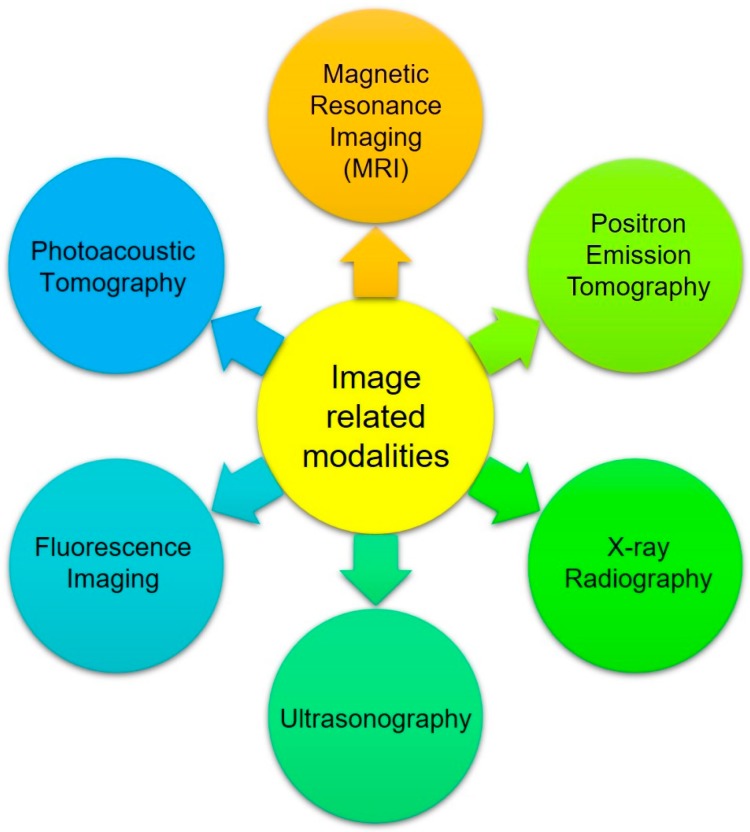
Image-related modalities.

Tetrapyrrolic-based macrocyclic systems are getting more attention in biomedical applications due to their various advantageous features like low cytotoxicity in the absence of light, tunable photophysical properties, such as absorption and emission wavelength, superficial derivatization, and superior tumor uptake of these chemical entities [36]. The use of porphyrins and their metal derivatives in molecular imaging and biomedicine is an interdisciplinary field, and significant growth has been observed in this area in the 21st century. Over the last few decades, due to the development of imaging devices and instruments and chemistry of imaging probes, significant interest and growth have been observed in the field of in vivo medical imaging. Now a day’s medical imaging is taking benefits from numerous modalities as shown in Figure 3.

MRI was first used in 1977 to visualize the human body, since then it has been the most extensive diagnostic imaging technique. MRI being a non-invasive in vivo imaging technology, is considered one of the top medical imaging techniques. This technology can provide 3-D images and offers many advantages over other modalities such as the absence of ionizing radiations and capability to provoke both anatomic and physiologic information. In earlier studies, tetrapyrrolic-based contrast agents have been used for the detection, diagnosis, and treatment of the defective tissues [27,37]. However, the development of a single multifunctional compound is highly desired to permit meditation in a single inspection of initial phase tumors and their growth process, drug action and pharmacokinetics [38,39]. MRI technology is based on the absorption of pulses of radiofrequency by a body when it is placed in a magnetic field. The fundamental principle of MRI is that when a magnetic field is allowed to pass through the tissue protons, after absorbing some of the electromagnetic energy, they will be transmitting the rest of the electromagnetic energy. The amount of the transmitted energy is dependent on the number of protons in the tissue, its environment, and mobility. The basic criteria for an MRI contrast agent is to affect the radiofrequency pulses. Specific coordinated metal ions can interact freely with the biological system, and with water molecules in the tissue and have the ability to influence the radiofrequency pulses. Generally, contrast agents are of two categories, which are paramagnetic and superparamagnetic materials [40]. Highly stable metal complexes as magnetic resonance imaging contrast agents have been produced and administered to patients for enhancing the contrast between the diseased and healthy tissues [41]. Gadolinium-based contrast agent in which gadolinium is tightly bound to a high-affinity organic ligand has been used for MRI examinations. Moreover, manganese is a promising metal ion for clinical applications because of its superior contrast enhancing properties. Manganese-based contrast agents have been prepared by insertion and tightly binding of manganese into the cavity of tetraarylporphryins. Researchers have prepared expanded porphyrins which are also known as texaphyrins and their metal complexes for potential applications in MRI [42,43,44]. 

Due to the advent of non-invasive in vivo imaging technique, the number of scientific publications in this field is significantly increasing. Among the different in vivo medical imaging techniques, MRI is undoubtedly one of the most studied, because very high-quality images of tissues can be obtained in a non-invasive way [38,45]. In the last few decades, scientists have tried to develop contrast agents for MRI technology. In this respect, tetrapyrrolic structures including porphyrins and metalloporphyrins are most commonly studied and their inherent attraction for tumor localization they have tempted many researchers headed for their potential application as contrast agents in MRI [37,46,47]. The development of new and more effective contrast agents for MRI is essential for convinced differences between healthy and diseased tissue. In 1971, Damadian [48], for the first time, used MRI technology to differentiate between normal and defective tissues. 

For clinical adaptations there are many restrictions on the molecular structure of the contrast agent, i.e., the contrast agent must be safe in use, and should be excreted from the body in case if it has a deleterious effect on body, should be easily soluble and essentially give best quality diagnostic images in a short span after administration into the body of patient [49]. The images can be produced by utilizing longitudinal and transverse relaxation time represented by T_1_ and T_2_, respectively. The MRI image of the inborn body tissue can be obtained via excitation of nuclei of the hydrogen atom of water, which is naturally present in the tissue of an organism, with contrast in the image resulting from the image obtained due to alteration in the tissue density. There are several clinically-approved contrast agents, but unfortunately, no one is commercially available for malignant neoplastic diseases. As a consequence, there is increasing attention in the preparation of more efficient contrast agents, comprising the use of porphyrins and metalloporphyrins [36,50,51].

There are many other factors which are crucial for the designing of contrast agents, like the effect of temperature, concentration, the strength of the magnetic field, kinetic and thermodynamic stability, binding of water molecules to the metal porphyrin complex, and its exchange rate with the bulk water [52]. Therefore, in synthesizing of contrast agents for MRI depend on the metal complexes having paramagnetic metals, alongside with appropriate groups capable of enhancing the relaxivity values and functionalities under investigation. The metal porphyrins complexes are kinetically inert, and there is the low possibility of release of metal ions from the porphyrin cavity during MRI investigations. Moreover, the stability of metalloporphyrins lowers the risk of toxicity, for example, fibrosing disorders and neurotoxicity caused by gadolinium and manganese, respectively [53,54].

**Figure 4:**
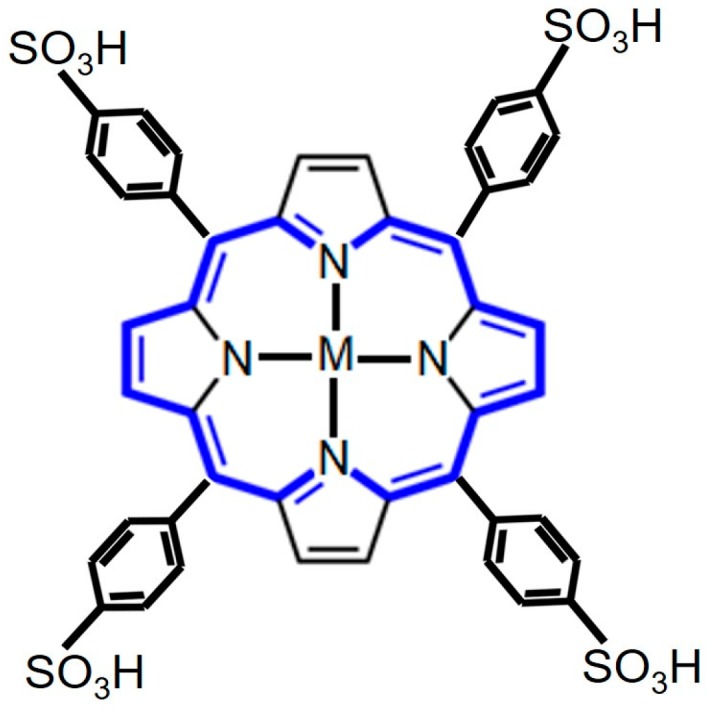
Typical structure of *meso*-sulphonatophenyl porphyrin which can be used as a contrasting agent for MRI (M = Cu(II), Fe(III), or Mn(III).

In 1984, for the first time, the tetrapyrrolic-based macrocycles were used as contrast agents in MRI. At that time metals like copper, iron, and manganese were inserted separately into the cavity of *meso*-tetrasulphonatedphenyl porphyrin (TPPS_4_) as illustrated in Figure 4. Additionally, their effect on the spin relaxation rate of water, which is represented by (1/T_1_), was determined. Among all the studied metalloporphyrins the manganese(III) TSPP_4_ complex was the best possible contrast agent with an r_1_ value of about 10.4 mM^−1^ s^−1^ and copper(II) proved a poor contrast agent with an r_1_ value 0.14 mM^−1^ s^−1^ [52]. In another study, Zou et al. [55] prepared C60-manganese porphyrin and observed higher r_1_ value as compared to 5-(4-aminophenyl)-10, 15, 20-tris(4-sulfonatophenyl) manganese(III) porphyrin. In conclusion, regarding the use of porphyrin and metalloporphyrins as promising MRI contrast agent, it can be concluded that, if the size of metal center is comparable with the size of porphyrin cavity then stable metalloporphyrins toward demetallation can be applied in vivo imaging techniques. Moreover, the efficiency of metalloporphyrins as a contrast agent can be enhanced by structural modification to optimize the relaxation time. Most importantly, the future of MRI technique must be based on intriguing molecules having multiple functionalities for excellent tissue contrast and tissue penetration.

## 4. Applications of Porphyrins and Metalloporphyrins in Photodynamic Therapy of Cancer

**Figure 5:**
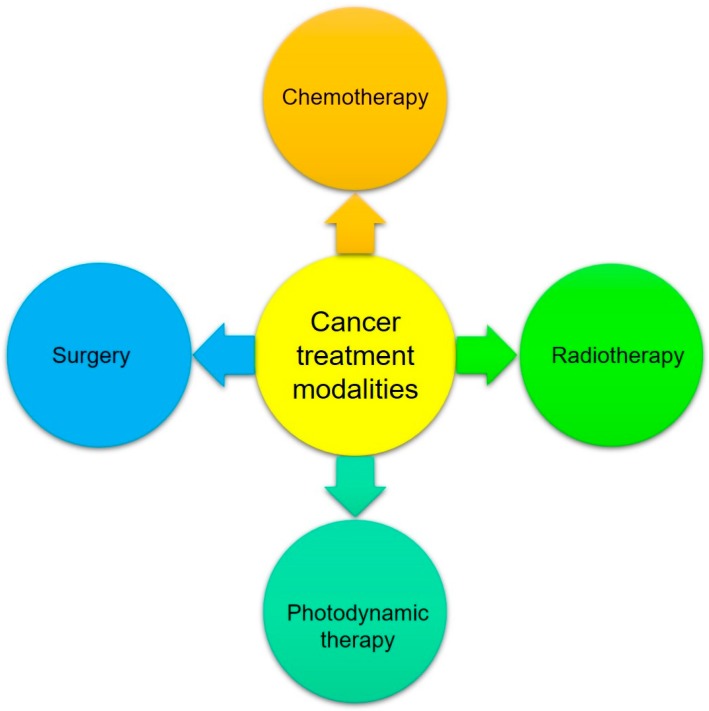
Fundamental and significant cancer treatment modalities.

Cancer is one the fastest-growing lethal diseases that human beings are facing all over the globe, and is supposed to be the second leading cause of death, having been responsible for 15% of all deaths [56,57]. The formation and growth of abnormal cells in the body may be initiated by various factors such as inherited mutations, chemicals, harmful radiations, weakness of immune system etc. [58,59]. The fundamental cancer treatment modalities are shown in Figure 5. However, all these treatments are associated with severe side effects to the patients and lengthy recovery times.

On the contrary, PDT is favorable to terminate abnormal cell growth and destroy the malignant tumor without harming to the healthy body tissues. PDT has numerous merits over other cancer therapies: it is insignificantly toxic and invasive, it can be applied to places where surgery is not possible, and it can be used for solid cancers of the skin, breast, prostate, etc. In addition to the application of photodynamic therapy in oncology, it is also advantageous for cardiovascular and other infectious diseases. Photodynamic therapy is a slightly invasive and encouraging treatment procedure to cure cancer. A perfect photosensitizer should be free from carcinogenic effect, photoactive at a wavelength between 600 and 790 nm, have high purity and stability, and sensitive and selective uptake in abnormal cells [60,61].

**Figure 6:**
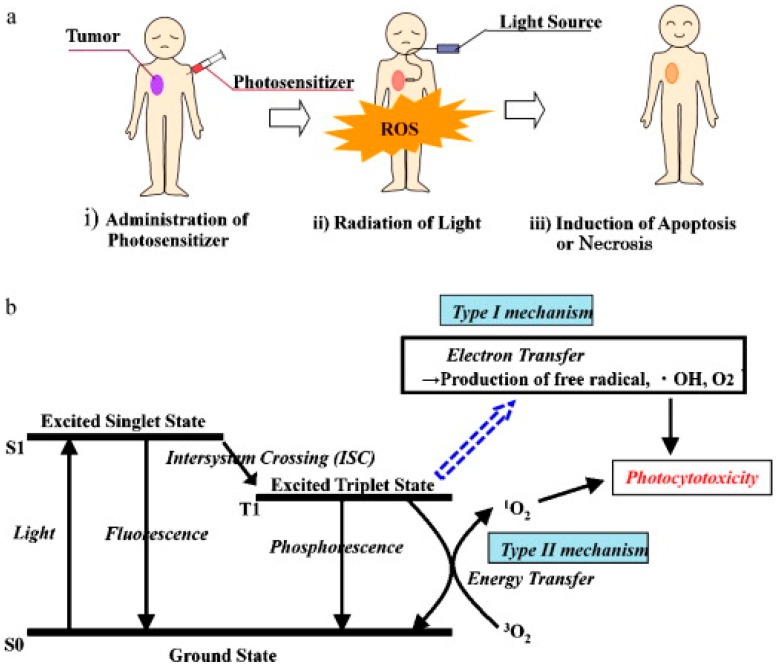
PDT mechanism: (**a**) profile of PDT treatment; (**b**) generation of excited states and reactive oxygen species (ROS). Reproduced from Yano et al. [62], with permission from Elsevier.

**Figure 7:**
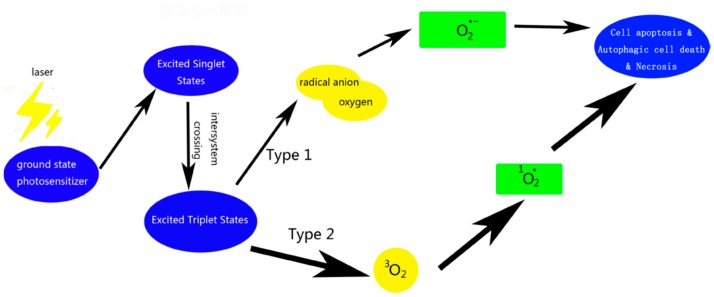
Activation of PS and generation of ROS involved in PDT. Reproduced from Kou et al. [66], an open-access article distributed under the terms of the Creative Commons Attribution License 3.0 (CC BY 3.0).

The general profile of PDT treatment is shown in Figure 6 [62]. In conventional PDT procedure, an appropriate dose of the photosensitizer is injected into the bloodstream of a patient. After a suitable time, the photosensitizer which accumulates in malignant cells is followed by irradiation with light of definite wavelength, a red light or the light with wavelength 550–800 nm is suitable for PDT of deeper tumor tissue. The light below 500 nm is not ideal for deeper tumor tissue. Photodynamic activity is initiated by the absorption of a photon by photosensitizer and followed by various radiative and non-radiative processes and may result in degradation or oxidation of biomolecules [62]. Molecular oxygen present in the tissue plays a vital role in the propagation of molecular damage resulting in tissue destruction and cell death. Hence, the photodynamic therapy induces cell necrosis by generating ROS. There are two types of photodynamic reactions: type I and type II. In type I processes, the excited state of the photosensitizer generally acts as photo-oxidant, oxidizing cellular substances, i.e., biomolecules like amino acid and DNA bases to form radicals or radical ions [62,63]. In type II process, as a result of interaction between electronically excited photosensitizer which is in triplet state with the molecular oxygen (^3^O_2_) yields highly active singlet oxygen (^1^O_2_) which react with many biological molecules including lipid, proteins, and DNA leading to cancer cells [29,63,64]. The transfer of energy from excited state to PS to oxygen and generation of singlet oxygen (^1^O_2_) is very efficient [29,65]. All the steps involved in the generation of ROS as shown in Figure 7 [66], except the generation of biomolecule-based radicals, and excited state of the photosensitizer will oxidize a biomolecule rather than oxygen to form superoxide.

Until now four generations of porphyrin-based sensitizers have been prepared, but each generation of PS suffers from drawbacks. For example, photofrine derived from hematoporphyrin a first generation photosensitizer and had been clinically approved worldwide for the treatment of various type of skin, lung, and gastric cancer to a certain degree, but, unfortunately, it suffers from many drawbacks. Firstly, it undergoes the formation of oligomers. Secondly, it has a long wavelength of absorption which is not appropriate for deep tissue penetration and more adverse effects of toxicity. There is an ample prerequisite to address all the shortcoming of porphyrins to realize these compounds to use for proficient PDT use to cure cancer [66].

## 5. Porphyrins and Metalloporphyrins for Drug Delivery

**Figure 8:**
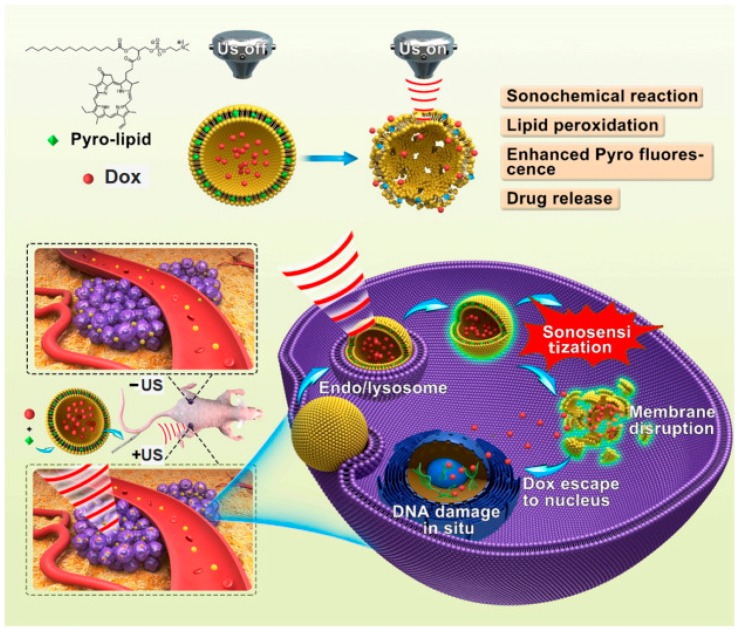
Representation of sonoactivatable, Dox-loaded porphyrin-phospholipid-liposome (Dox-pp-lipo) for anti-tumor treatment. Reproduced from Wang et al. [67], with permission from Elsevier.

The delivery of drug at a specific point in the body has vital importance in disease treatment. More recently, researchers are developing the strategies which enable the co-delivery of drug and gene for cancer treatment and other disease therapies. In particular, photochemical internalization, which is a novel technology that can be employed for the site-specific release of medicine within in target cells. Recently, Wang et al. [67] reported an enhanced drug delivery using sonoactivatable liposomes with membrane-embedded porphyrins. The release mechanism of sonoactivatable doxorubicin (Dox) loaded porphyrin-phospholipid-liposome (Dox-pp-lipo) for anti-tumor treatment is shown in Figure 8 [67]. Ma et al. [68] prepared star-shaped polymer and a porphyrin material which was good at drug delivery at a cellular level.

Porphyrin-based metal-organic frameworks (MOFs) are hybrid materials and have been widely studied for drug delivery applications. Porphyrin-based MOF’s have tunable porous structures enable materials to have high drug loading ability, adaptable functionality, and biodegradability. This type of porphyrin-based material can deliver the drug at a controllable rate and is a promising candidate for drug delivery in therapeutic applications [69]. The application of metalloporphyrins in drug delivery may minimize the complications of direct administration of drugs like dose quantity and different side effect due to the nonspecific distribution of drug [70]. MOF based materials have the potential to address the basic challenges for an efficient drug delivery carrier like stability and compatibility within the physiological environment. Moreover, different types of interactions like hydrophobic interactions, hydrogen bond and van der Waal’s force between drug and MOF allows a sustained and adjustable release of drug from metalloporphyrins with stimuli-responsive. The factors like temperature and pH can be helpful in controlling the release rate of a drug. Researchers have tested the MOF in oral drug delivery [71,72]. Lin et al. [69] synthesized porphyrin-based MOF as an oral drug carrier. They selected methotrexate drug which was absorbed into the pores of MOF by diffusion. The synthesized material showed high drug loading capacity, sustained release, and controlled pH-responsive release for the drug [69].

Despite simple MOF, modified porphyrin (m-porphyrin) at the nano level has been considered as a drug carrier and targeted delivery of two poorly-soluble anticancer drugs, i.e., tamoxifen and paclitaxel (taxol), to a specific area [73]. Dong et al. [18] synthesized a porphyrin-incorporated hydrogel containing a four-arm copolymer for dual fluorescent drug delivery system. Kejik et al. [74] propose a covalent attachment of a therapeutic protein to a drug delivery system which is a cyclodextrin conjugated with a metalloporphyrin. They synthesize a drug system which was based on a combination of Zn-porphyrin conjugated with cyclodextrin. This system allows combined cell targeted chemotherapy and immunotherapy; this coordination assembly displayed therapeutic advantage when tested in a human carcinoma [75]. Furthermore, Gardella et al. [76] reported a drug delivery system based on a porphyrin, poly(L-lactide), and graphite; this material proved extremely promising in drug delivery filed.

## 6. Role of Porphyrins and Metalloporphyrins in the Determination of Ferrochelatase in Bone Marrow

In living organisms, the attachment of metal ion into the cavity of porphyrin macrocycle is catalyzed by a group of enzymes known as chelatases. For example, ferrochelatase catalyzes the insertion of the ferrous ion into the porphyrin cavity for the synthesis of heme and magnesium, and cobalt chelatases catalyze the attachment of Mg and Co in the synthesis of chlorophyll and vitamin B_12_ [77]. Due to the various functions of heme, ferrochelatase plays a critical role in human health. Human genetic defectiveness affecting this enzyme may result in a disease known as erythropoietic protoporphyria. Human ferrochelatase represents the convergence of tetrapyrrole synthesis in the presence of iron and plays a vital role in complete body iron metabolism [78]. To determine ferrochelatase activity, various assays have been developed in the last decades. One of the most commonly used assay for ferrochelatase involves the use of porphyrins, ferrous ion substrates, and the spectrophotometric measurements of the synthesized heme, this method can detect the very low amount of protoheme formed [79]. Another assay measures ferrochelatase activity by monitoring the disappearance of porphyrin, because under these conditions porphyrin and heme show an isosbestic point which indicates the conversion of substrate into product. The progress of the reaction can be monitored by using steady-state absorption techniques [80]. Shi and Ferreira [81] developed spectrofluorometry assay for determining ferrochelatase activity using the physiological substrates ferrous iron and protoporphyrin IX. In contrast to heme, the product of the ferrochelatase-catalyzed reaction, protoporphyrin IX is fluorescent and, therefore, the progress of the reaction can be monitored by following the decrease in protoporphyrin fluorescence intensity. This continuous fluorimetric assay detects activities as low as 0.01 mmol porphyrin consumed min^−1^ [81].

Roberts [82] reported an HPLC method to estimate the activity of ferrochelatase in the human liver cell. In this method partially homogenate of liver cells was incubated in the presence of Co(II) and mesoporphyrin. After a fixed time, the porphyrin was extracted into an ethyl acetate-acetic acid mixture, and the ferrochelatase activity was investigated by the rate of utilization of mesoporphyrin [82]. Cornah [83] developed a fluorimetric method assay for ferrochelatase that employs cobalt and deuteroporphyrin in place of natural substrates and measures the decrease in fluorescence of deuteroporphyrin. In summary, porphyrin-based molecules may provide a sensitive method to measure ferrochelatase activity. There is an ample need to design of materials based on porphyrins which can detect a low level of ferrochelatase activity in biological samples and porphyria patients.

## 7. Bio-Imaging Applications of Porphyrins and Metalloporphyrins

The field of medical imaging is developing at a significant rate with the advent of noninvasive in vivo technologies, like fluorescence imaging (FI), to speed up drug design and development progression. Medical imaging techniques can point out size, shape, morphology of organs in the body and guide to develop a suitable medical procedure. Tetrapyrrole-based macrostructures and their derivatives or phthalocyanines are most widely studied compound in biomedical applications [84], and their ability to accumulate in different types of cancer cells. Radiolabelled metalloporphyrins have also been widely studied for bio-imaging applications. The basic condition is radiolabelled metalloporphyrins is that it must have positron emission nuclides like ^13^N, ^18^F, and ^11^C with a high percentage of positron decay. Some other nuclides like ^64^Cu, ^52^Fe, ^111^In, and ^67^Ga can also be used [85]. The radiolabelled chlorin was able to detect and monitor the progression of tumors [86]. Moreover, Fazaeli et al. [87] has prepared ^68^Ga-fluorinated porphyrin complex as a possible PET imaging agent. It has been reported that metalloporphyrins have displayed exciting tumor-avid activity both in vivo and in vitro. Gadolinium motexafin complex is a unique photosensitizer that has been used in radiotherapy of brain cancer [88]. Chen et al. [89] reported a porphyrin conjugate in which a photosensitizer is linked with dye for tumor imaging applications.

There is an immense need to develop a single probe which can simultaneously be applied to several imaging modalities. For this purpose, multiple bio-imaging probes have been designed that permit combination of many imaging techniques like fluorescence imaging and computed tomography, positron emission tomography (and single-photon emission computed tomography, etc. Luo et al. [90] synthesized tetranuclear gadolinium(III) porphyrin complex as a theranostic agent for multimodal imaging and photodynamic therapy. They incorporated a Gd(III)-based chelate moiety to tetraphenyl porphyrin, considering both functions of Gd(III)-based chelate in MRI and porphyrin in PDT are likely achieved in a combined compound. A porphyrin and a porphyrin zinc complex as a dual-function molecular imaging platform for MRI and fluorescence imaging sensing has been reported [91].

## 8. Major Limitations and Recommendations

Porphyrins and other tetrapyrrole-based macrocyclic photosensitizers as therapeutic modalities have been of interest for over many decades, yet the applicability of all the discussed compounds in this review as clinical treatments of abnormal cell growth still need significant improvement. Similarly, the utility of metalloporphyrins in biomedical science have been recognized over many decades, but none of the discussed structures of metalloporphyrins have yet been approved for clinical applications.

Porphyrins and their metal derivatives can be used as an MRI contrast agent, but the following concerns should be addressed while designing potential MRI contrast agents. First of all, the development of contrast agents are specifically designed for high magnetic field applications because nowadays clinical scanners are becoming more common. Secondly, biological distribution is also crucial while developing an MRI. It should localize in one type of tissue to highlight the pathology of the targeted tissue. Moreover, it is recommended to design the metalloporphyrins which should be able to retain an MRI contrast agent suitable for higher magnetic field strength and high relaxivity. Another crucial consideration for a contrast agent is toxicity which is considered to depend on the kinetic and thermodynamic stability of the metal porphyrin complex. Therefore, the critical goal in the development of a contrast agent depends on the design and evaluation of porphyrin-based compounds bearing a paramagnetic metal ion along with suitable moieties capable of increasing relaxivity values at higher magnetic field strength. Furthermore, the properties of metalloporphyrins can be improved either by electronic or chemical modification, or by conjugation with biomolecules of higher molecular weight. Interaction with bovine serum albumin can increase the relaxivity of porphyrins and their metal complexes. It is also recommended that the brominated metal porphyrin complexes are preferable because they exhibit increased water-proton relaxivities as compared to non-brominated metalloporphyrins. Another possible route for developing an effective contrast agent is asymmetrical functionalization of sulfonatophenyl porphyrin.

The following are the recommendations for an ideal photosensitizer for PDT. At first, a photosensitizer should be able to strongly absorb in the phototherapeutic window (650–800 nm), low fluorescence quantum yield, excellent stability, and a long lifetime of excited triplet state and high yield of singlet oxygen. Moreover, the structure of a photosensitizer should be amphiphilic which should facilitate its accumulation in the cell membrane. The skeleton of porphyrin is hydrophobic which can be transformed into amphiphile by the introduction of hydrophilic group. Very large substituents like polymers and peptides can also be substituted on the porphyrinic macrocycle to be considered for the design of a photosensitizer for PDT. Metal complexes with bacteriochlorin may be used as a photosensitizer for the generation of ROS. Application of nanomaterials may be helpful in the selective localization of the photosensitizer and may enhance the ROS generation. Furthermore, in recent years, the targeted drug and gene delivery to a specific area has gained prime importance in the medical-related research and development sector. Porphyrin-based MOFs have been studied in recent years as a potential host for drug delivery. The ideal candidate for drug delivery must have a porous structure, low toxicity, high drug load capacity, and good biocompatibility.

The future of imaging techniques should be based on the molecular species with reasonable spatial resolution, maximum tissue penetration, multimodal imaging capability, safety, stability against demetallation, and maximum paramagnetic character. All these desired properties can be achieved by chemical modification of metalloporphyrins or by preparing nanoparticles by conjugating porphyrins on an appropriate support.

## 9. Concluding Remarks and Perspectives

Research in biomedical sciences is rapidly growing and intervened with clinical success. New approaches are being developed to improve the consequence of diagnostic and therapeutic uses. Porphyrins have a highly extended conjugated π-electron system which imparts aromatic behavior, and its limited size cavity is suitable to bind numerous kinds of metal ions. Porphyrins and metalloporphyrin-based materials have fascinating and tunable photoredox, photophysical, and photochemical properties that can be exploited in various biomedical applications. Porphyrins can absorb light strongly in the visible region, and this energy can be used for photophysical and photochemical reactions. This review article showed how these promising properties can be beneficial for fabricating novel and operative materials for MRI, PDT, drug delivery, diagnostic imaging, and other clinical applications.

In conclusion, looking at the application of metalloporphyrin-based materials as a potential contrast agent for magnetic resonance imaging the following points should be considered, first of all, for the enhanced relaxivities, safety for in vivo application, stability against demetallation under physiological conditions, and biological distribution. Therefore, to address all the requirements for a compelling contrast agent material, it should be comprised of a stable metal complex having metal with maximum paramagnetic character and suitable substituents capable of enhancing the relaxivity values. Moreover, nanoparticle systems conjugated with porphyrin derivatives are a promising candidate to achieve high relaxivity values and biodistribution. Additionally, due to the tailored photophysical and photochemical properties of tetrapyrrole-based macrocycles, their metal complexes have been recognized to be efficient photosensitizers in PDT. To consider a compound as a useful photosensitizer, it should be pure with intense absorption in the visible or near-infrared region, and produce singlet oxygen with high quantum yield. It must be non-toxic and able to eliminate from the body rapidly to avoid any harmful effect on surrounding tissues. Water-soluble porphyrins and their metal complexes are favorable options that can fulfill all the requirements mentioned above and can be used as photosensitizers in PDT. The effect of size, electronic configuration of the metal center, and peripheral substituents on photophysical properties are essential factors in designing an efficient photosensitizer for photodynamic therapy. The efficiency of a porphyrin-based material can be improved by a combination of different substituents into the metalloporphyrin frameworks. There are still many challenges, like the toxicity, biological distribution, and photostability of photosensitizers, which can be addressed by tuning the photophysical features of the tetrapyrrolic-based material. Porphyrin-based MOFs and porphyrin-grafted silicon nanoparticles have been widely studied for drug delivery and other clinical applications. Metalloporphyrin nanoparticles can be modified with different functional groups through axial coordination following the nanoparticle formation for diverse functions. In summary, through sophisticated design, multifunctional characteristics of porphyrins and metalloporphyrin-based materials can be adjusted to an optimal level to be used for diagnostic imaging and biomedical applications. For future development more work is still needed to advance porphyrin-based material to the clinical stage, and it is important to have collaboration between chemists, biologist, and clinicians.
